# Exacting Responses: Lack of Endocrine Cephalic Phase Responses Upon Oro-Sensory Exposure

**DOI:** 10.3389/fendo.2018.00332

**Published:** 2018-06-13

**Authors:** Marlou P. Lasschuijt, Monica Mars, Cees de Graaf, Paul A. M. Smeets

**Affiliations:** ^1^Division of Human Nutrition and Health, Wageningen University and Research, Wageningen, Netherlands; ^2^Image Sciences Institute, Brain Center Rudolf Magnus, University Medical Center Utrecht, Utrecht, Netherlands

**Keywords:** texture, taste, insulin, pancreatic polypeptide, ghrelin

## Abstract

Oro-sensory exposure (OSE) to food plays an important role in the regulation of food intake. One proposed underlying mechanism is the occurrence of cephalic phase responses (CPRs). CPRs include the pre-digestive endocrine responses induced by food-related sensory input. Yet, whether OSE duration or sweetness intensity affects CPRs is unknown. The objective of this study was to determine the independent and interactive effects of oro-sensory duration (chewing) and stimulation intensity (sweetness) on endocrine CPRs and satiation. Eighteen males (22 ± 2 years, BMI 22 ± 2 kg/m^2^) participated in a 2 × 2 randomized study with a control condition. Each session participants performed modified sham feeding (MSF) with one of the four gel-based model foods. During the control session no MSF was performed. Model foods differed in chewing duration (hard or soft texture) and sweetness (low or high intensity). During each session, eight blood samples were collected up till 25 min after MSF onset. Subsequently, food intake from an *ad libitum* lunch was measured. No typical CPR was found for insulin, pancreatic polypeptide (PP), and ghrelin. However, the overall PP response was 1.1 times greater for the hard sweet MSF condition compared to control (*p* = 0.02). Overall ghrelin responses were 1.1 times greater for the hard model food compared to the soft model food conditions (*p* = 0.003). These differences in endocrine response were not associated with differences in food intake at the subsequent meal. Exploratory sub-analysis of the responsive insulin curves showed that after 2.5 min of MSF the hard texture model foods insulin concentrations were 1.2 greater compared to the soft texture. These findings indicate that texture hardness and sweetness increase the overall PP response and that MSF on hard texture increases the overall ghrelin response compared to soft texture model foods. However, MSF on model foods does not lead to a typical CPR. This study, among others, shows that there are major dissimilarities in the endocrine responses to food stimulation between individuals. This emphasizes the importance of considering cephalic responders and non-responders. More research is needed to understand CPRs in relation to food texture and taste properties.

## Introduction

The current food environment drives a positive energy balance leading to a growing number of obese and overweight people worldwide (1, 2). Knowledge about the mechanisms by which food intake is controlled is key in finding solutions to this problem (3).

Food intake starts with the breakdown of food in the mouth; this process depends on the structure, the flavor, and the palatability of the food (4–7). Besides food qualities, oral processing is determined by the individuals’ anatomy, such as mouth size, strength of the jaw muscle (masseter), and automated eating behavior characteristics such as bite size and chewing rate (8–10). Intake of a food is inhibited when the aforementioned food and individuals’ characteristics lead to a slow ingestion rate (11). Several physiological mechanisms have been suggested to cause this effect, among which the oro-sensory exposure (OSE) to food (12, 13).

Oro-sensory exposure can be defined as the release of nutrients, odor, and taste molecules from the food matrix in the mouth during oral processing (14). Foods that do not require chewing before the bolus is ready to swallow induce only limited OSE, whereas foods that do require chewing enhance OSE (7). Beverages, for example, can be ingested in a relatively short time and therefore the oral-residence time and thus OSE is limited. This may be a reason why energy-containing beverages suppress appetite and energy intake less compared to equicaloric solid foods (15, 16).

Oro-sensory exposure is necessary for the initiation of cephalic phase responses (CPRs) (16). The cephalic phase is the first phase of digestion, including all physiological, endocrine, and autonomic responses stimulated by cephalic phase sensory input such as taste, smell (OSE), and the sight of food (17, 18). The putative function of these anticipatory responses to food is to optimize digestion and to minimize the impact of meals on homeostasis (19–22). Initial cephalic responses signal stimulation of food intake, while continued sensory stimulation may induce satiation (18). Cephalic phase signals include the production of saliva and endocrine responses in insulin, pancreatic polypeptide (PP), and ghrelin.

A lack of, or diminished cephalic phase responsivity as a result of decreased OSE through rapid food consumption, has been suggested to affect physiological and psychological processes, such as glucose homeostasis, metabolism, and food reward and satiety systems in the brain (12). In both animal (23, 24) and human studies (25), it has been shown that these changes are related to decreased appetite responses and weight gain (26). OSE may therefore play a key role in the regulation of food intake through the induction of CPRs (10, 27, 28).

However, the importance of the duration and intensity of the OSE in order to induce CPRs is unknown. OSE duration affects the CPR as shown by studies of Teff et al. where cephalic responses of insulin and PP were found to mixed nutrient foods and solid foods but not to liquids (29, 30). CPR could also be enhanced through taste (31). This is shown by a study of Just et al. where an increase in insulin plasma concentration was found upon oral cavity stimulation with a sweet sucrose and saccharine solution but not for any of the other taste qualities (32). In addition, Teff found a PP response to mixed nutrient sweet and salty foods with a higher magnitude of the response found for the sweet foods (29). This indicates that sweet taste may have a specific role for nutrient signaling that aids in controlling food intake and food digestion through CPRs (33).

Taken together, OSE can be varied in duration and intensity which may affect CPRs and consequently food intake. However, it is not known if, and to what extent OSE duration and taste intensity induce cephalic responses. Therefore, the main objective of this study was to determine the independent and interactive effects of oro-sensory duration (chewing) and stimulation intensity (sweetness) on the endocrine CPRs and subsequent food intake. To investigate this, we performed a 2 × 2 factorial randomized crossover study with control condition. Endocrine responses were measured under conditions in which OSE duration (hard and soft texture) and taste intensity (low sweetness and high sweetness) were varied. We expected a higher peak of the endocrine responses (insulin, PP, and ghrelin) for increased OSE magnitudes (i.e. hard texture, long chewing duration, and high sweet intensity) and a consequently lower food intake.

## Materials and Methods

### Subjects

The study was performed at Wageningen University, The Netherlands. Subjects were recruited from the surroundings of Wageningen using flyers and posters. In addition, emails were sent to persons in a database of volunteers who previously had expressed an interest in participating in nutrition studies. Healthy male subjects between 18 and 35 years old with a BMI between 18.5 and 25 kg/m^2^ were recruited. Subjects had to eat three meals a day around the same time, and were excluded if they followed an energy-restricted diet or if they had gained or lost >5 kg of body weight during the past 2 months. Subjects were also excluded if they had dental pathologies, chewing, swallowing, or eating difficulties, self-reported taste or smell problems, braces or dentures, and when they used medication. In addition, they were not allowed to participate if they were high-restrained eaters according to the Dutch Eating Behavior Questionnaire (DEBQ): score >2.9 (34). Personnel and thesis students of the Division of Human Nutrition were omitted from participation. During the information meeting, subjects rated the model foods that were used in this study on liking. Subjects were excluded from participation if they disliked one of the model foods (defined as score <4 on a nine point hedonic Likert scale) or had a stronger preference for one of the model foods compared to the others (defined as >2 point difference on a nine point hedonic Likert scale).

Potential subjects were invited to a training visit at Wageningen University. During the training subjects practiced with modified sham feeding (MSF) by repeatedly chewing and spitting out the entire model food bolus upon the moment they would normally swallow. In addition, height and body weight were measured and a research nurse measured the subjects’ Hb value (finger prick) and judged the forearm veins for suitability to place an intravenous cannula. Subjects were excluded after the training session if their Hb value was not within 8.1–11 mmol/l or when the research nurse decided that the veins of the forearm were not suitable for placement of the intravenous cannula. In addition, participants were excluded if they had a recovery rate <85% of the dry weight (see Modified Sham Feeding) of the model food. This 85% cut-off point has been used in previous studies (35).

The study was approved by the Medical Ethical Committee of Wageningen University, The Netherlands (ABR: NL5682408116) and registered in the Dutch trial register under NTR5870 (http://www.trialregister.nl). All subjects signed informed consent prior to the training. Subjects received a financial compensation for their time and effort.

**Figure 1:**
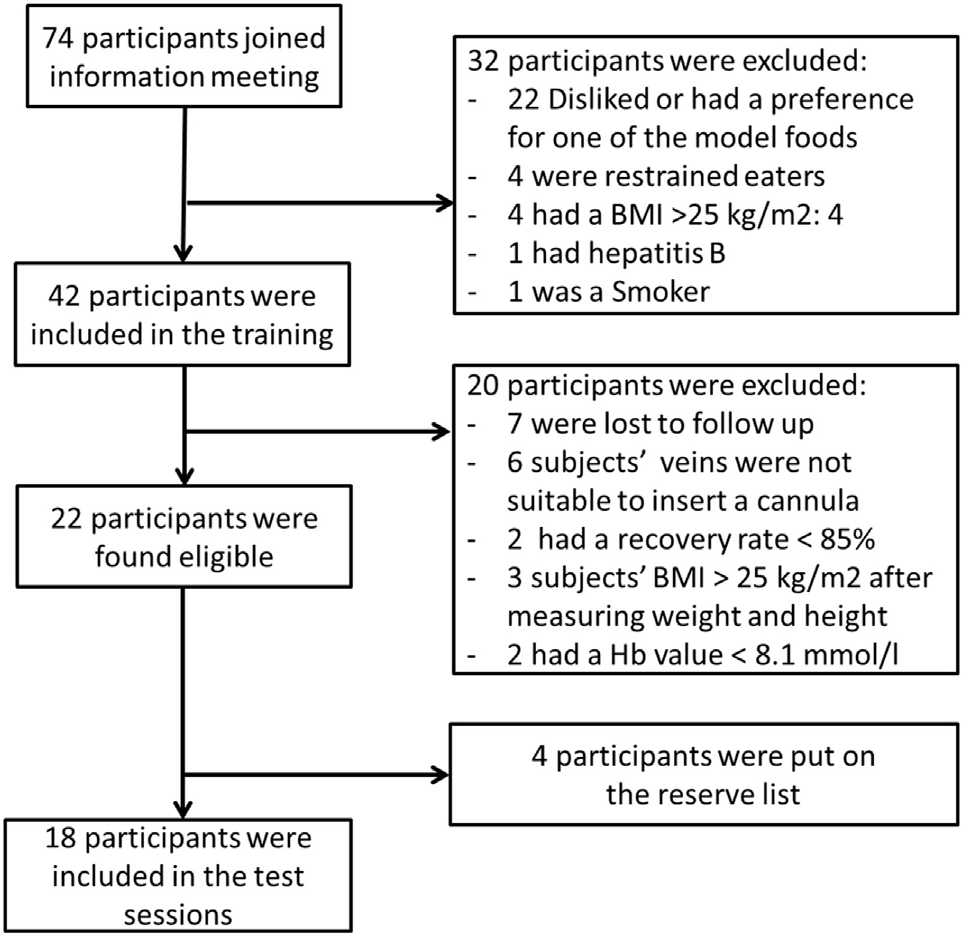
Flow chart of the in- and exclusion of subjects.

Prior to the study, sample size calculations showed that a minimum of 18 subjects was needed to show an effect of 10% between treatments. For this calculation, we assumed a variation coefficient of 15% incremental area under the curves (IAUC) for the control condition and 20% IAUC for the experimental conditions. In addition, a correlation of within person measures of *r* = 0.6 was assumed (22, 36). Power was set at 1-β = 0.80 at a significant concentration of α = 0.05. In total, 74 subjects joined the information meeting of which 42 subjects were found eligible; however, seven were lost to follow-up. Finally, 35 subjects joined the training session of which 22 subjects were eligible to be included in the test sessions, see Figure 1. Finally, 18 were invited for the study.

The subjects that participated in the study were on average 22 ± 2 years old, had a mean (±SD) body mass index of 22 ± 2 kg/m^2^ and an average DEBQ restraint score of 1.9 ± 0.5 (34).

### Experimental Design

The study had a 2 × 2 factorial randomized crossover-design with a control condition. During the four treatment visits subjects sham fed one of the four types of gel-like models foods. Model foods were formulated to have two levels of oral processing time (soft or hard texture), and two concentrations of sweetness (low or high) (see Model Foods). During the control visit, subjects did not sham feed or eat anything. During each visit, blood samples were taken at fixed time points and after the last blood sample collection, subjects received an *ad libitum* lunch meal. Conditions were randomized, following a Latin square Williams design with 1-week washout in-between visits.

### Experimental Procedures

The evening preceding the test session subjects were instructed to eat the same meal of their choosing to avoid confounding effects of previous food consumption. Subjects were instructed to eat the meal between 18:00 and 20:00 h after which the subject was not allowed to eat or drink anything except water until the next morning. Subjects received two 500 ml cartons of a milk-based breakfast drink (banana, kiwi, strawberry flavored “goedemorgen fruitontbijt” Vifit Wageningen, The Netherlands, Friesland Campina) to drink until fullness 4 h prior to their arrival time. Subjects were instructed not to eat or drink anything after their breakfast drink and to bring back the cartons. The breakfast drink cartons were covertly weighted to determine intake (average intake of the breakfast drink was 662 ± 258 ml). Subjects were also instructed to avoid high-intensity exercise (everything besides regular speed walking and biking) and alcoholic drinks 24 h preceding the test and to use the same mode of transport every time they visited. To control for compliance subjects were asked to keep a diary of their food and beverage consumptions and physical activity the day before the test session.

Half an hour prior to the start of the test sessions, an intravenous cannula was placed in the antecubital vein by a trained research nurse. Blood drawings were done in a quiet room by a trained research nurse.

Subjects were provided with 15 pieces (of 7 g each) of the model foods at *t*2.5 (baseline sample) which they had to smell. After that subjects started chewing on the model foods, starting to chew was defined as *t* = 0. Blood samples were taken at time points *t* (min), *t*2.5 (smell), *t*0 (start MSF), *t*2.5, *t*5, *t*10, *t*15 (stop MSF), *t*20, and *t*25. Subjects regularly filled in an appetite questionnaire (see Appetite Ratings and Well-Being).

**Figure 2:**
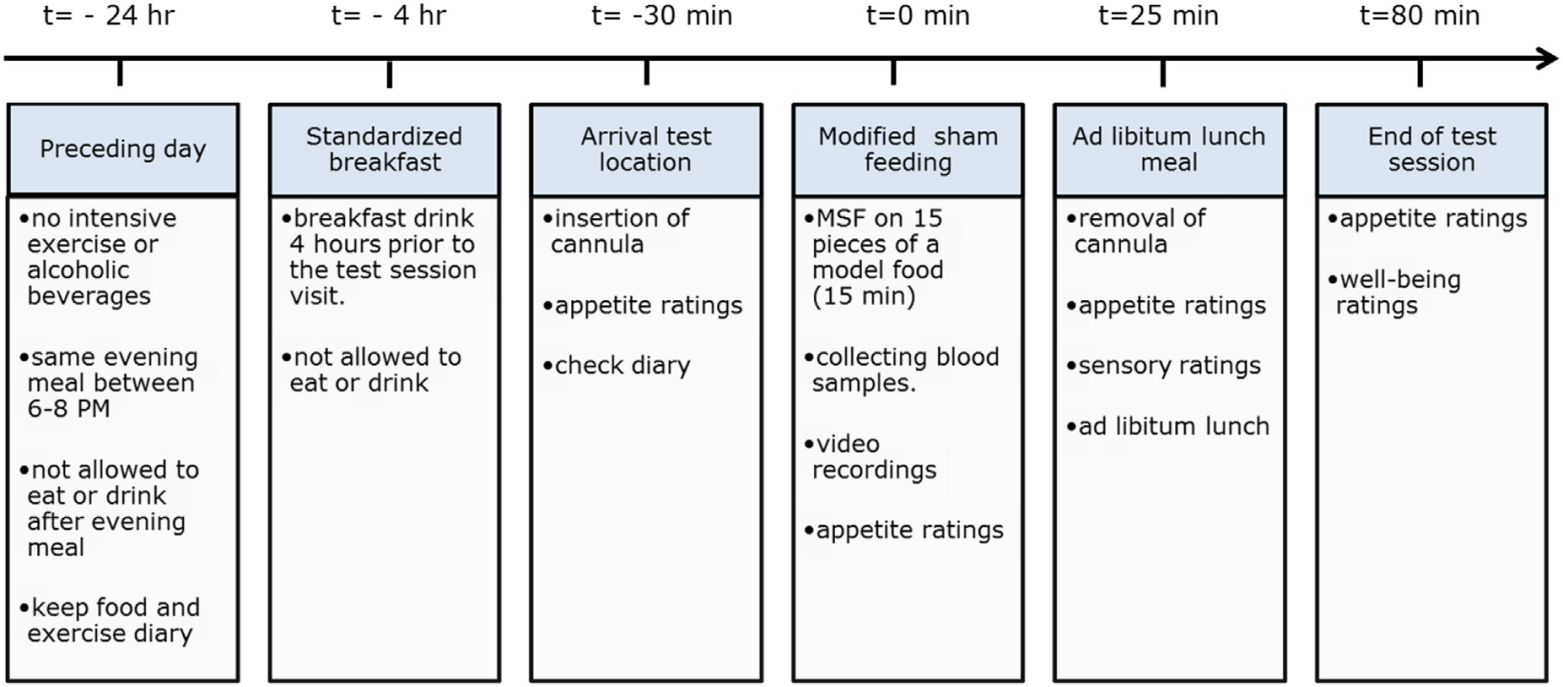
Overview of experimental procedures.

During the sham feeding session, subjects were recorded with use of a webcam to determine the number of chews and chewing duration. After the sham feeding, part of the test session subjects were provided with an *ad libitum* lunch meal to measure intake as a measure of satiation. For an overview of the entire test session procedure, see Figure 2.

#### Model Foods

Strawberry flavored gel-based model foods similar to our previous study (37) were used. Ingredients used were cream (30% fat, AH Basic), sunflower oil, strawberry flavored pudding powder (“Jelly,” Dr. Oetker) and water. To manipulate oral processing time, the hardness of the gels was altered by added thickening agents, that is, carrageenan (type CHP-2) and locust-bean gum (LBG). Thickening agents were added in the following amounts: 0.22/0.22 wt% carrageenan/LBG for the soft and 0.66/0.88 wt% carrageenan/LBG for the hard model food.

In our previous study, we were unable to increase the difference in perceived sweetness between the low and sweet version of the model foods without changing the liking of the model foods by using a non-caloric sweetener. For the present study, multiple pilot studies were performed to enhance the sweetness difference between the low and high sweet model foods by adding table sugar. We were able to establish a (mean ± SEM) 16 ± 2 mm difference (rated on a 100 mm VAS, *p* < 0.001) in sweetness between the low and high sweet model foods, with no difference (*p* = 0.90) in sweetness between textures (soft and hard) (mean ± SEM) 0.3 ± 2.3. By adding table sugar next to the non-caloric sweetener, we were able to keep palatability of the model foods equal (mean difference ± SE 1.2 ± 2.6, *p* = 0.80). The added calories (table sugar) in the high sweet conditions were thought to be negligible as subjects sham fed the model foods (chew and spit) and did not ingest, in contrast to our previous study (37).

In the final study model, foods significantly differed in sweetness concentration [*F*_(3,51)_ = 13.6, *p* < 0.001] but the different types of model foods were equally liked (mean ± SE, 100-mm VAS: 56 ± 3). Participants did not have a preference for a texture type (*p* = 0.38) or sweetness concentration (*p* = 0.23). The hard and soft texture low sweet variants were significantly less sweet 49 ± 2 compared to the high sweet variants 66 ± 2 (*p* < 0.001). Perceived hardness significantly differed between texture concentrations [*F*_(3,51)_ = 56, *p* < 0.001], mean ± SEM perceived hardness of the hard model food was (58 ± 2) and of the soft model foods (21 ± 2). The average number of chews per model food piece significantly differed between texture variants [*F*_(1,17)_ = 138.8, *p* < 0.001] but not between taste concentrations (*p* = 0.64). Participants chewed on average 31 ± 2 times on the hard model food and 20 ± 2 times on the soft model foods. Chewing duration also differed between texture variants [*F*_(1,17)_ = 95.5, *p* < 0.001] and not between taste concentrations (*p* = 0.76). Average chewing duration on the hard model foods was 22 ± 1 s and on the soft model foods 16 ± 3 s. These model food characteristics are similar to those found in our previous study (37). The ingredients, energy content and macronutrient composition of each of the model foods can be found in Table 1.

**Table 1:** Energy and macronutrient content of the model foods per 100 g.

Model food	Soft low sweet	Soft sweet	Hard low sweet	Hard sweet
Energy (kJ/kcal)	786/188	869/208	803/192	882/211
Protein (g)	0.8	0.8	0.8	0.8
Fat (g)	13.6	13.6	13.6	13.6
Carbohydrate (g)	15.6	20.5	16.5	21.4

#### Modified Sham Feeding

Subjects were instructed to chew on the model food and to expectorate the entire bolus upon the moment they would normally swallow. This chew and spit method is called MSF and has been used in previous studies (35, 38, 39). With this technique, subjects are orally exposed to the model foods without ingestion. Subjects were trained to MSF during the training session (see Subjects).

Subjects received 15 identical pieces of 7 g of the model foods which they had to “chew and spit.” To avoid the confounding effects of “eating” rate rather than number of chews and chew effort on the metabolic and hormone response, subjects received instructions on when to start chewing a new model food piece. After the maximum amount of time had passed (40 s), they were, however, instructed to expectorate the model food if not previously done. The maximum amount of time of 40 s was determined based on a previous study performed with the same model foods (37). Compliance to the instructions for MSF not swallowing the (pieces of) model food was determined by analyzing the dry mass of model food that was spat out. Recovery percentage was calculated by dividing the dry mass of the expectorated boli by the dry mass of the model foods, in line with the method used by Wijlens et al. (35).

In this study, the mean recovery percentage was (mean ± SE) 92.7 ± 0.8%. In grams this means that on average 2.4 ± 0.3 g of the model foods was swallowed. Per model food type this was 92.0 ± 0.7% (2.2 ± 0.2 g swallowed) for soft low sweet; 89.3 ± 0.8% (3.9 ± 0.3 g swallowed) for soft high sweet; 94.1 ± 0.4% (1.7 ± 0.1 g swallowed) for hard low sweet; and 95.6 ± 0.4% (1.6 ± 0.1 g swallowed) for hard high sweet. This is in line with other studies that also report recovery percentages between 89 and 97% (35, 40–42).

#### Blood Collection and Plasma Analysis

Glucose concentrations were measured by collecting a blood sample *via* the cannula with a syringe and was directly measured using a blood glucose meter (FreeStyle Freedom Lite).

Insulin samples were collected in 3 ml lithium heparin-coated vacutainer tubes and placed on ice immediately after acquiring. Insulin samples were centrifuged at 1,300 *g* for 10 min at 4°C. Plasma samples for insulin measurements were stored at −25°C until analysis at hospital “De Gelderse vallei” in Ede, The Netherlands. The detection limit of the analysis ranged from 2 to 300 mU/l with an intra assay CV of 2.2% and inter assay CV of 5.7%.

Pancreatic polypeptide blood samples were collected in 2 ml EDTA tubes, stored on ice after acquiring, and centrifuged at 2,500 *g* for 15 min at 4°C. Plasma samples were stored at −80°C until analysis. PP was analyzed by using Human PP Elisa kit (Millipore) with a detection range of 12.3–3,000 pg/ml and a intra assay CV of 3.3% and a inter assay CV of 4.9%.

Ghrelin samples were collected in 2 ml EDTA tubes with added AEBSF blocker (Pefabloc^®^ SC) to a final concentration of 1 mg/ml. Samples were stored on ice after acquiring and centrifuged at 2,500 *g* for 15 min at 4°C. Ghrelin plasma samples were acidified to a final concentration of 0.05 N before storage.

Human ghrelin total ELISA (Millipore) was used to analyze total ghrelin concentrations with a detection range of 50–5,000 pg/ml and a intra assay CV of 1.11% and a inter assay CV of 5.18%. In case of measured values below the detection limit, the lowest detectable concentration of the analysis was used for data analysis.

#### Oral Processing Characteristics

To measure oral processing characteristics, subjects were video recorded during each session. A webcam (resolution 640 × 280 pixels) was positioned in front of the subject (face-on) where the lower frame was in line with the shoulders, and the upper frame above the top of the cranium and the sides at shoulder width. Subjects were instructed to limit their head movements. Video recordings were analyzed with the use of Observer Noldus XT 11. Behaviors of interest were chewing duration (s) and number of masticatory cycles (number of chews). From these variables, chewing frequency was calculated by dividing the number of chews by the total chewing duration.

#### Saliva Excretion

Fluid content of the model foods was calculated by subtracting the dry weight of the model food from the total weight of the eaten model foods. In addition, the fluid content of what was spat out was determined by subtracting the dry weight of the spat out bolus from the total amount that was spat out. Saliva content was then calculated by subtracting the water content of the model foods from the fluid content of the expectorated boli (43).

#### Appetite Ratings and Well-Being

**Figure 3:**
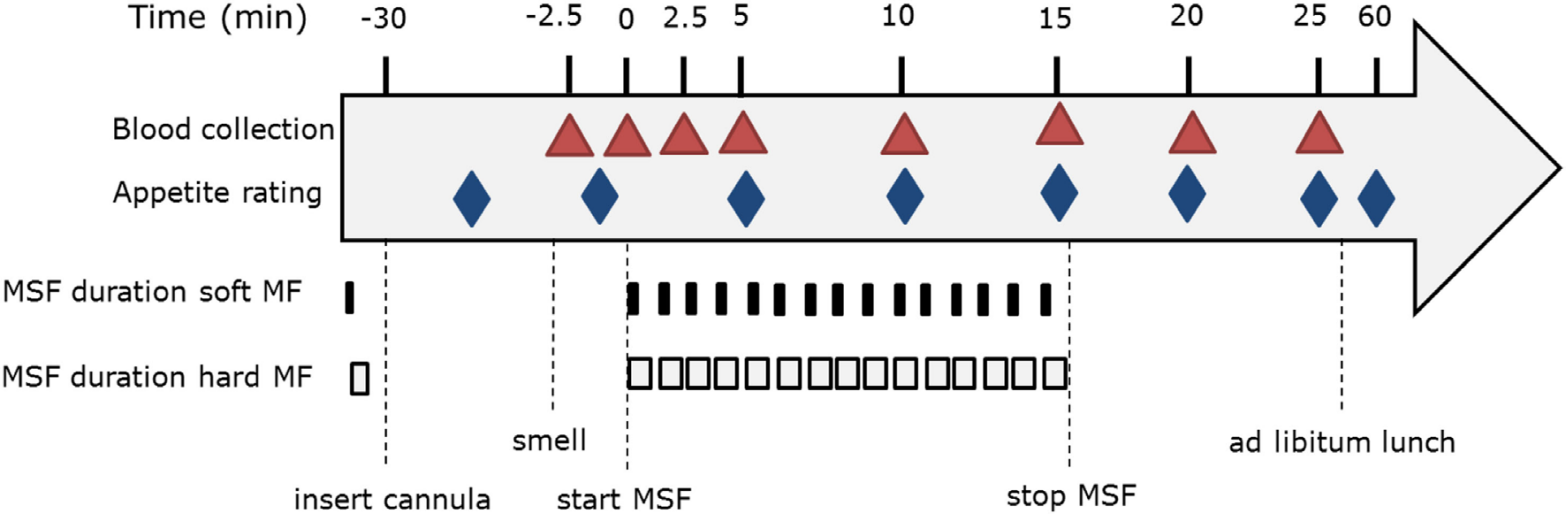
Modified sham feeding (MSF) procedure overview including blood sample collection and appetite ratings.

Subjects rated satiety feelings, well-being, and model food characteristics on a 100-mm VAS anchored with “not at all” at 10 mm and “extremely” at 90 mm, see Figure 3. Subjects rated hunger, fullness, and desire to eat (DTE) at every time point. Due to time limitations; thirst, DTE, DTE sweet, DTE savory, prospective consumption, and nausea were rated at *t* = 20, 25, 60 min (Figure 3). Subjects rated their well-being after insertion of the cannula, at the same time of each blood sample collection and directly after the *ad libitum* lunch meal. Liking of the model food was rated at *t* = 5 (liking of the taste) and after the sham feeding session at *t* = 30. After the indwelling cannula was removed participants ate a model food piece and rated additional model food specific parameters; liking, DTE the gel, sweetness and hardness.

#### Food Intake

Directly after the MSF experiment, the cannula was removed and subjects received an *ad libitum* lunch meal. The lunch meal consisted of sandwiches made from two slices of whole bread cut in four small pieces of bread with different types of topping; full fat cheese, ham, apricot jam, or hazelnut spread. The amount of bread toppings was determined in such way that breads with different toppings were iso-caloric (50–52 kcal per quarter piece). To make sure there was a surplus of sandwiches (*ad libitum*), subjects receive 200% of a normal portion size of bread for each of the different toppings. Based on an average lunch energy intake of 20% of the daily energy need for men (2,500 kcal), subjects should be offered five servings (two slices of bread on top of each other with topping in between) equal to 20 sandwich quarters of each topping.

During these meals, subjects were seated in separate booths and were given 100 ml water with their lunch meal, that they were instructed to drink completely in sips between bites over the course of their lunch meal. They were instructed to eat until pleasantly full, and not to talk to each other. The weight and number of bread rolls was covertly weighed and counted to determine intake.

### Statistics

Statistical analyses were performed using SAS (version 9.3; SAS Institute Inc., Cary, NC, USA). Results are presented as mean ± SEM unless otherwise stated. *p*-Values <0.05 were considered statically significant. To test endocrine concentrations and appetite scores, mixed model ANOVA (PROC MIXED) was used. In this model, texture, taste, time, and their interactions were added as fixed factors, the repeated statement was used to indicate the repeated measures over time per subject. Compound symmetry was used as a covariate structure. Normality of the data was checked by visual inspection. Outcome variables that were not normally distributed (insulin, PP, and ghrelin) were log10 transformed before analyses. For these variables, geometric means and ratios with 95% CI are reported.

Outcomes were tested for an order and baseline effect and were found to be significant covariates. Therefore, outcomes (insulin, pp, and ghrelin) were corrected for order and baseline concentrations by adding these two variables as a covariate. Tukey correction was used to compare means between treatments and Dunnett correction to compare means of treatments with control.

In addition, IAUC were calculated for all endocrine outcomes by means of the trapeziual rule and analyzed by means of a mixed model with conditions (texture and taste) as fixed factor, subject as random variable and baseline AUC as covariate. This model without covariate was also used to calculated differences in intake during the *ad libitum* lunch meal and differences in secreted saliva during MSF between treatments.

## Results

### Cephalic Phase Responses

#### Glucose

**Figure 4:**
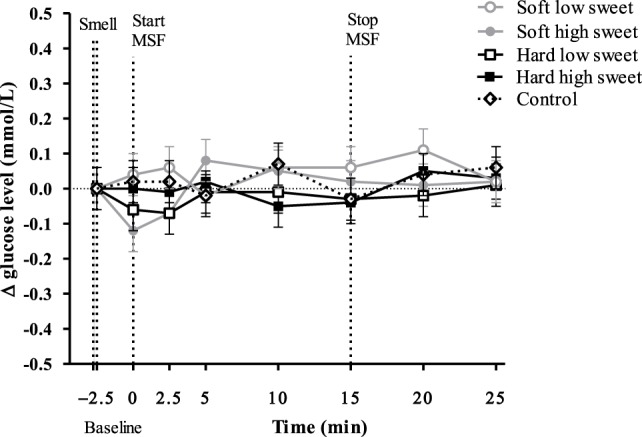
Estimated mean ± SEM Δ glucose concentration (mmol/l) from baseline over time for all treatments and the control condition. No significant differences were found between treatments or control condition at any time point (*p* = 0.96).

Glucose concentrations remained constant over time [*F*_(7,199)_ = 0.78, *p* = 0.61] with no differences between treatments and the control condition at any of the time points [*F*_(28,476)_ = 0.59, *p* = 0.96], see Figure 4.

#### Insulin

**Figure 5:**
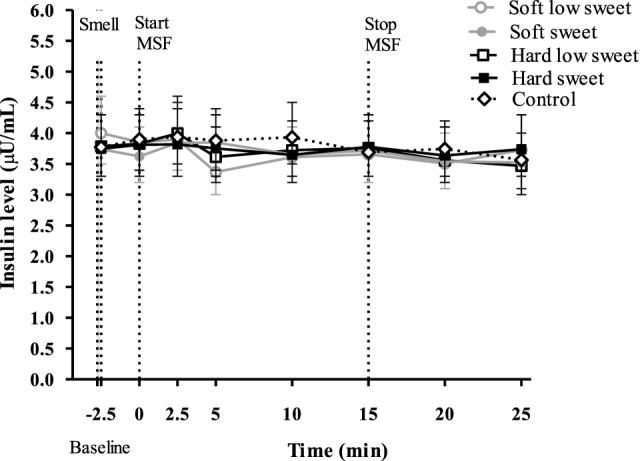
Geometric mean ± 95% CI insulin (μU/ml) concentrations over time per treatment and control condition. No significant differences were found between treatments and control condition at any of the time points (*p* = 0.97).

We did not observe an insulin peak during any of the study treatments and we found no significant differences between treatments or control condition at any of the time points [*F*_(28,310)_ = 0.53, *p* = 0.97], see Figure 5. Insulin concentrations changed over time [*F*_(7,199)_ = 2.6, *p* = 0.02], insulin levels at 5 min were 1.1 (95% CI: 1.0–1.2) times higher compared to insulin levels at 20 and 25 min. However, these differences in insulin concentrations between the time points could not be attributed to the texture [*F*_(7,119)_ = 0.22, *p* = 0.98] or taste [*F*_(7,119)_ = 1.12, *p* = 0.35] manipulations, see Figure 5. IAUC did not differ between treatments [*F*_(1,50_) = 0.16, *p* = 0.69] or between treatments and control condition [*F*_(4,67)_ = 0.72, *p* = 0.58].

#### Pancreatic Polypeptide

**Figure 6:**
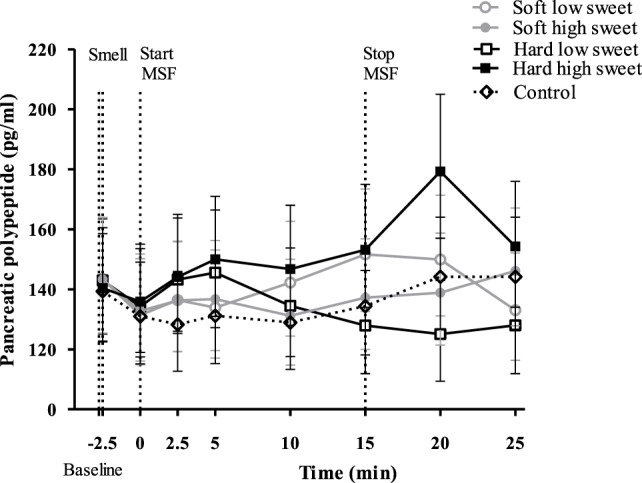
Geometric mean ± 95% CI pancreatic polypeptide concentrations (pg/ml) corrected for baseline concentration over time per treatment and control condition. No significant differences were found between treatments and control condition at any of the time points (*p* = 0.72).

Pancreatic polypeptide concentrations did not change over time [*F*_(7,119)_ = 1.03, *p* = 0.42], neither did the PP concentrations differ between treatments nor control condition at any of the individual time points [*F*_(28,476)_ = 6.18, *p* = 0.72], see Figure 6. However, the total PP response to the hard sweet MSF condition was 1.1 times greater compared to control, which was significant (95% CI = 1.0–1.2, *p* = 0.02). In addition, we found a significant texture/taste interaction effect on PP concentrations [*F*_(1,17)_ = 7.53, *p* = 0.01], the response for the low sweet hard texture model food was 0.9 times lower compared to the sweet hard texture model food curve (95% CI = 0.8–1.0, *p* = 0.019). These effects were, however, too small to show significant differences in IAUC between treatments [*F*_(1,50)_ = 2.6, *p* = 0.11] or between treatments and control condition [*F*_(4,67)_ = 1.0, *p* = 0.40].

#### Ghrelin

**Figure 7:**
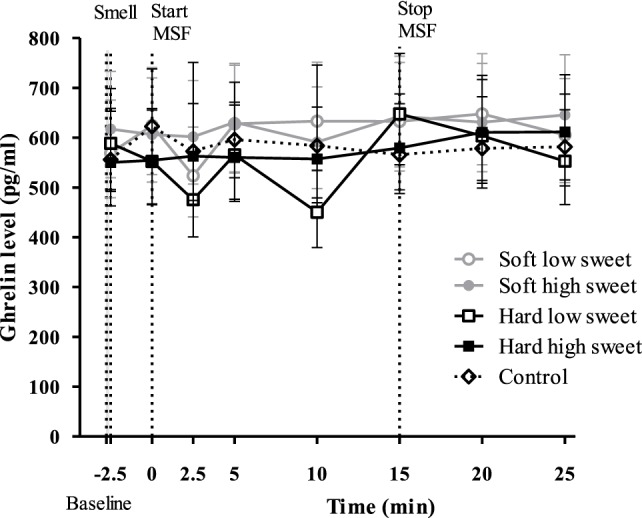
Geometric mean ± 95% CI ghrelin (pg/ml) concentrations corrected for baseline concentration per treatment and control condition. No significant differences were found between treatments and control condition at any time point (*p* = 0.63).

No significant changes over time were found for any of the treatments [*F*_(28,476)_ = 0.90, *p* = 0.63], see Figure 7. In addition, we did not find differences between treatments or control condition at any of the individual time points. However, we did find a significant texture effect combining all time points [*F*_(1,17)_ = 12.3, *p* = 0.003]. Ghrelin curves of the hard model food conditions were 1.1 times higher compared to the soft model food conditions (95% CI = 1.0–1.2, *p* = 0.003). However, these differences were too small to result in a difference in IAUC between the treatments [*F*_(1,50)_ = 0.27, *p* = 0.60] and control condition [*F*_(4,67)_ = 0.48, *p* = 0.75].

#### Saliva Excretion

**Figure 8:**
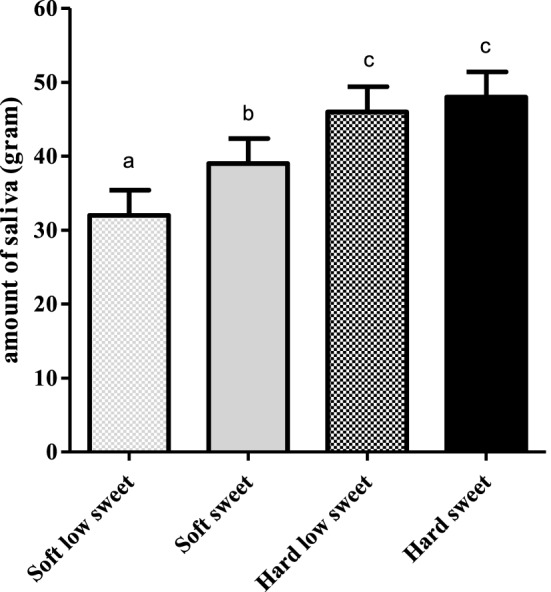
Estimated mean ± SEM saliva content (in grams) of expectorated boli per model food type. Bars with different letters are significantly different.

We found a significant effect of texture [*F*_(1,17)_ = 74.6, *p* < 0.001] and taste [*F*_(1,17)_ = 11.3, *p* = 0.004] but no interaction effect [*F*_(1,17)_ = 3.6, *p* = 0.08] on expectorated saliva, see Figure 8. When MSF on hard model foods participants expectorated 12 ± 1.4 g more saliva compared to MSF on the soft model foods (*p* < 0.001). In addition, MSF on a sweet model food led to a 5 ± 1.4 g increased expectoration of saliva compared to MSF on a low-sweet model food (*p* = 0.004).

#### Exploratory Findings: Cephalic Phase Insulin Responders and Non-Responders

In line with the approach of Dhillon et al. (44) for each condition (including control), curves were considered responsive when insulin concentration increased at *t* = 2.5 from baseline (*t* = −2.5). Based on this, 54% of the curves were classified as responsive and 46% of the curves as non-responsive, see Table 2. At a subject level, 33% could be considered responders, defined as having three or four responsive curves out of the four treatments.

**Table 2:** Percentage of responsive and non-responsive curves per treatment and control condition.

	Soft low sweet	Soft sweet	Hard low sweet	Hard sweet	Control	Total percentage
% responder	15	15	29	20	22	54
% non-responder	25	25	12	20	18	46

**Figure 9:**
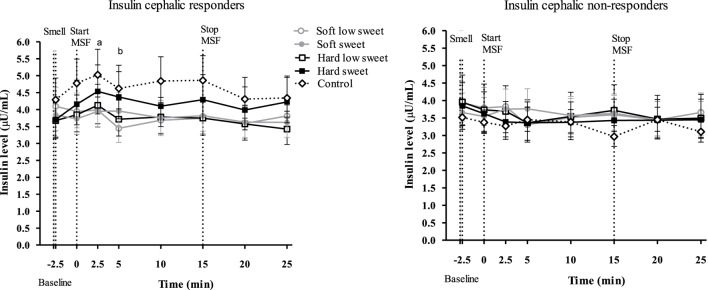
Geometric mean ± 95% CI insulin responder and non-responder curves. Letter “a” indicates a significant texture effect (*p* = 0.049) and “b” indicates a significant sweetness effect (*p* = 0.005).

For the responsive model, we found a significant texture [*F*_(1,13)_ = 4.84, *p* = 0.047], taste [*F*_(1,17)_ = 9.67, *p* = 0.006], time [*F*_(7,119)_ = 2.16, *p* = 0.042] and texture/taste interaction effect [*F*_(1,5)_ = 9.72, *p* = 0.026] on insulin concentrations over all time points. Analyzing insulin concentrations per time point we found a texture effect [*F*_(1,47)_ = 4.09, *p* = 0.049], 2.5 min after starting sham feeding. Insulin concentrations at 2.5 min were 1.2 times (CI% 1.0–1.4) higher for hard compared to the soft texture conditions (*p* = 0.049). In addition, we found a significant taste effect [*F*_(1,47)_ = 8.89, *p* = 0.005] after 5 min of MSF. Insulin concentrations were 1.2 times (CI% 1.1–1.4) higher for low sweet compared to the sweet model foods (*p* = 0.005), see Figure 9.

In addition, a sub-analysis was performed of PP and ghrelin concentrations based on the insulin classification of responder, non-responder curves. For the PP non-responder and responder curves, we did not find any effects (all, *p* ≥ 0.3) or differences between treatments and control (*p* = 0.06).

For the ghrelin non-responder and responder curves, we found a significant effect between the hard sweet curve and control curve (*p* = 0.009). For the ghrelin responder curves, we found a texture effect over all time points (*p* = 0.007), hard texture was generally lower compared to the soft texture but not at any specific time point.

### Appetite and Well-Being

**Figure 10:**
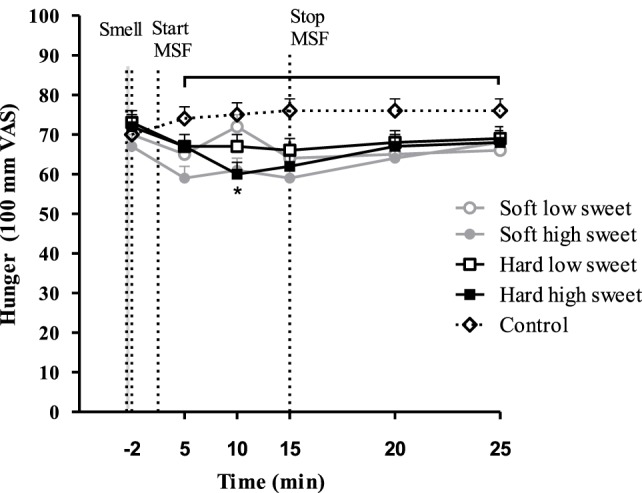
Estimated mean ± SEM hunger changes over time per treatment and control condition. Significant differences were found between treatments and control indicated by the bracket (*p* < 0.05). *Significant taste effect (*p* = 0.003).

**Figure 11:**
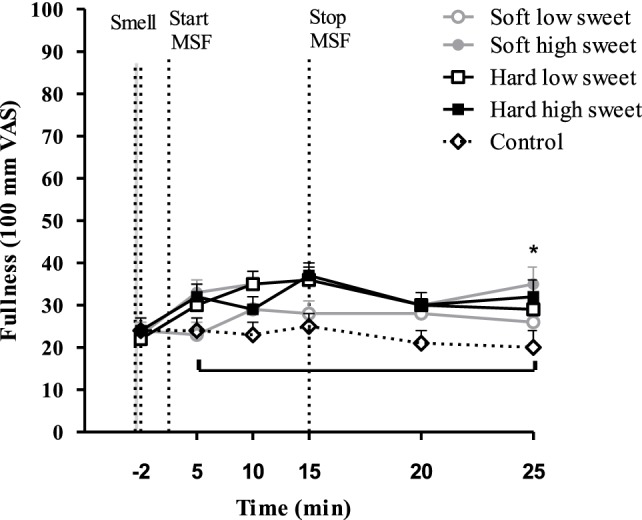
Estimated mean ± SEM fullness changes over time per treatment and control condition. Significant differences were found between treatments and control indicated by the bracket (*p* < 0.05). *Significant taste effect (*p* = 0.04).

Hunger feelings over time were significantly lower for the hard high sweet, soft high sweet, and low sweet model foods compared to control, see Figure 10. After 10 min of MSF subjects felt less hungry for the sweet compared to the low sweet model foods (mean ± SEM 9 ± 3 difference, *p* = 0.003). Fullness ratings showed a similar trend; fullness over time was scored higher after MSF on the hard sweet and low sweet model foods and the soft sweet model food compared to control (Figure 11), significant time points are indicated by the bracket (*p* < 0.05). A small but significant taste effect was found 10 min after the MSF period; subjects felt more full after the high sweet compared to the low sweet MSF treatments (mean difference = 6 ± 4, *p* = 0.04). Prospective consumption ratings post MSF on the sweet soft (*p* = 0.03) and hard (*p* < 0.007) model food differed from control. Prospective consumption ratings were lower (mean ± SEM 8 ± 2) 10 min after MSF comparing the low (*p* = 0.03) and high sweet (*p* = 0.012) soft model food with the control condition. No significant changes (pre to post) were observed between treatments or control condition for DTE sweet or savory. There were no differences in participants’ well-being, dizziness, feeling to faint, nausea, and thirst (before, or after MSF) between treatments and the control session (all, *p* > 0.32).

### Effect of MSF on Food Intake

**Figure 12:**
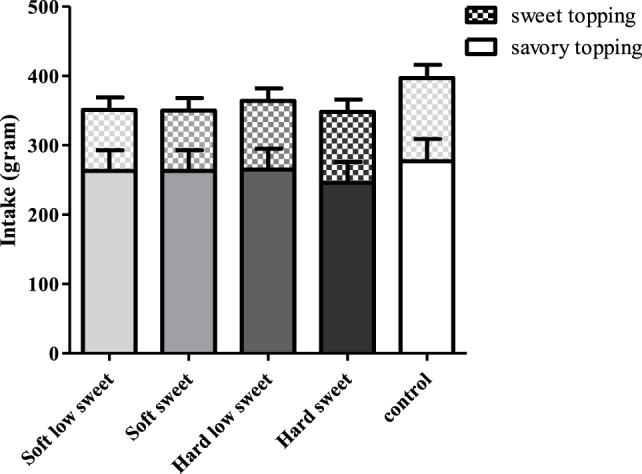
Estimated mean ± SEM post modified sham feeding (MSF) lunch intake (in grams) per model food type. No significant differences were found.

There was no effect of texture [*F*_(1,17)_ = 0.03, *p* = 0.87] or taste [*F*_(1,17)_ = 0.06, *p* = 0.81] or an interaction effect of texture and taste on intake [*F*_(3,17)_ = 0.06, *p* = 0.81], see Figure 12. In addition, we did not find a significant difference between intake after the MSF sessions and control condition [*F*_(4,68)_ = 0.35, *p* = 0.84]. In addition, no texture, sweetness, or interaction effect on intake of the sweet toppings (jam and hazel nut spread) or savory toppings (ham and cheese) was found.

## Discussion

We investigated the independent and interactive effects of OSE duration (chewing) and stimulation intensity (sweetness) on endocrine CPRs, and subsequent food intake. In order to study this, we successfully developed a model food system that aid in studying realistic texture and taste manipulations on endocrine responses. The model foods used in this study were equally liked but differed in sweetness level and chewing duration.

The findings of this study show that, insulin levels at 5 min after starting to MSF were 1.1 times higher compared to insulin levels at 20 and 25 min, but these differences between time points could not be attributed to the texture or taste manipulations. In addition, the overall PP response of the hard sweet MSF condition was 1.1 times greater compared to control and a significant texture/taste interaction effect was found. The PP curve of the low sweet hard texture model food was 0.9 times lower compared to the sweet hard texture model food curve. Comparing the total ghrelin curves, we found that the overall ghrelin response of the hard model food conditions was 1.1 times greater compared to the soft model food conditions. No typical cephalic phase peak response was found for any of the endocrine outcome measures. In addition, the found differences are small relative to the variation and would therefore be unlikely to have a physiological effect.

During MSF, participants expectorated 12% more saliva during MSF on the hard compared to the soft model foods. In addition to this texture effect, participants expectorated 5% more saliva when MSF on the sweet compared to the low-sweet model foods. Subjective appetite ratings showed that, in general, subjects felt fuller and less hungry after MSF compared to no MSF. These differences in appetite ratings were, however, not reflected in subsequent food intake during the *ad libitum* lunch meal.

These outcomes are not in line with our hypothesis as we expected to find a typical cephalic peak/spike in the endocrine responses (insulin and PP) and an increase in ghrelin concentrations for increased OSE magnitudes (i.e., hard texture, long chewing duration, and high sweet intensity) and a consequent decrease in food intake.

Our observed lack of a cephalic phase insulin response (CPIR) is in contrast with other studies that did find a CPIR upon sensory stimulation (36, 44–48). Based on conclusions of Teff et al., we expected a typical insulin response of approximately 25% above baseline or 1% of a normal postprandial insulin release within 2–4 min after sensory stimulation, returning back to baseline concentrations by 8–10 min (36, 49). However, we observed no changes from baseline in this study for which there could be various explanations. Characteristics of the stimuli or food used to evoke the cephalic response are of great importance. The foremost important aspect is palatability; it is assumed that the amplitude of the CPIR depends on the palatability of the stimuli used (50). Studies that have found a CPIR used highly palatable food stimuli such as apple pie (30) while the model foods used in this study were rated as neutral (average liking score was 56 mm on 100-mm VAS).

In addition to palatability, the stimuli should consist of multiple sensory modalities to induce insulin secretion as argued by Zafra et al. (12). This explains the lack of findings in studies where simple nutrient solutions (12, 30, 51) and sweet tablets (52) were used as stimuli. Only one study has shown an increase in insulin plasma concentration upon sucrose and saccharine oral cavity stimulation with a liquid (45 s swirl and spit method) (Just et al.) (32). However, this insulin response was smaller (1.5 μIU/mL rise from baseline for sucrose and 0.9 μIU/mL rise from baseline for saccharine) compared to the 25% above baseline; corresponding to a 2 μIU/mL increase, described by Teff et al. (36, 49). This emphasizes the importance of texture and taste qualities for the induction of detectable cephalic insulin response based upon which we expected to find a cephalic response in this study. Power and Schulkin argues that the association between insulin response and palatability is more important than nutrients, similarly this may also hold for the sensory modalities being more important than nutrients (22).

Besides the stimuli properties, subject characteristics determine the magnitude of the CPIR. The subjects included in this study were healthy and had a low, although healthy, BMI which could possibly explain the lack of CPIR; Teff et al. found that obese subject exhibit a larger CPIR compared to lean subjects (53). Besides BMI, there are several studies that report that there are cephalic phase responders and non-responders (44, 45, 54). In a recent study of Dhillon et al., insulin concentrations were measured during swirling of sweet drinks and MSF of sweet gelatin cubes. In this study, 64 overweight and obese subjects were included and a clear distinction could be made between responsive and non-responsive subjects. Over all treatments, 45% of the measured insulin curves were considered to be responsive (rise in insulin 2 min after stimulation). Insulin responses were mostly observed after exposure to sucrose in solid form (44). In addition, Bellisle et al. also failed to observe a CPIR in 12% of the subjects and Teff et al. found 50–75% of the subjects to be responders (45, 54).

Based on the classification of Dhillon et al. (44) in the present study, 54% of the insulin curves were considered responsive and 46% were considered non-responsive. Sub-analysis of the responsive curves showed an increased insulin concentration at 2.5 min after starting to MSF on the hard model foods, compared to soft. In addition, at 5 min an increased concentration for the sweet compared to the low sweet model foods was found. This indicates the importance of considering insulin responsive and non-responsive subjects when studying CPRs. However, the insulin responder, non-responder classifications were not confirmed by PP and ghrelin curves. This stresses the need of clear responder non-responder criteria for the different cephalic endocrines.

Although CPIR has been most extensively studied, PP is considered to show a more robust endocrine cephalic response that is not influenced by nutrients such as glucose but considered a vagal stimulation marker (36). PP increases 100% above baseline starting 10 min after the onset of a meal or after MSF, and concentrations remain elevated for another 30 min (55). Because PP responses are of a larger magnitude compared to insulin responses they are likely a better measure of graded CPRs to OSE (such as duration and intensity as investigated in this study). However, we also did not find a cephalic PP response. This is not in line with our hypothesis, as several other studies were able to detect a PP response in the absence of a CPIR (56, 57).

However, the lack of a cephalic PP response when MSF model foods is in line with the study findings of Mennella et al. that found no PP response when subjects MSF pudding, which is comparable in texture to the soft model foods used in this study (58). Our findings are also in line with findings of Teff et al. that found no difference in cephalic PP response between sweetness concentrations when subjects sham fed high sweet (unpalatable) and sweet (palatable) cream cheese crackers (29). In light of this finding, it is not surprising that we did not find a difference between sweetness concentrations that were within the palatability range and therefore did not differ largely in sweetness. This suggests that cephalic endocrine responses are not sensitive to small differences in sweetness levels.

Besides the lack of a CPIR and cephalic PP response, we also did not find an increase in ghrelin concentrations comparing treatments to control. Based on other studies we expected an increase in ghrelin concentrations suggesting an initial appetizer effect of MSF (59). In addition, we expected a decrease in ghrelin toward the end of MSF as we hypothesized that continued sensory exposure would lower ghrelin levels (25, 40, 59). The fact that we did not observe either of these ghrelin responses could be due to the mixed macronutrient content of the model foods as it is hypothesized that carbohydrate meals decrease ghrelin concentrations whereas fat and protein stimulate ghrelin secretion (25).

Saliva release is one of the earliest described CPRs, by Pavlov in 1910 (60) and has been documented since by various other studies (61–64). We observed a significant difference in the amount of saliva produced upon MSF the hard and sweet model foods compared to the soft and low sweet model foods. This is in line with previous reports that describe an up to fivefold increase of basal saliva release after 30 min of MSF a steak and French fries meal (65). The sensory receptors that are involved in saliva release are both chemical and mechanical (the movement of chewing stimulates saliva flow) (12). This can explain the differences found in saliva produced between texture and sweetness concentrations in our study. The act of chewing serves as a mechanical stimulant of the sensory receptors stimulating saliva flow. The fact that we found a sweetness effect on saliva production is in contrast with findings of Mattes and Pedersen et al. who showed that the macronutrient composition of the food determines the quality rather than the quantity of saliva. For example, sucrose and fructose stimulate amylase-rich saliva (12).

Compared to control, MSF decreased feelings of hunger and prospective consumption and increased fullness over the sham feed period up to 15 min. Differences between treatments and control were approximately 10 mm (100-mm VAS), which is modest but considered meaningful (66, 67). These findings are in line with previous studies that showed that prolonged oral stimulation alone suppresses hunger and increases fullness (35, 68). The differences in appetite ratings were not reflected in the amount eaten during the consequent meal, although, intake was 11% lower for the MSF conditions compared to the control condition but this was not significant. This in contrast with a study of Wijlens et al. that found a 15–19% decrease in intake after MSF cake for 8 min (35). Possibly, in our case, the total chewing duration was not long enough to affect intake significantly. A study of Mennella et al. also did not report an effect of MSF pudding on intake at a consequent meal (58). Few MSF studies have investigated the effect on subsequent food intake; most studies had a preload design with real food intake where subjects were instructed to chew each bite for a fixed number of time after which they measured intake of a meal (20, 69, 70). For example, a study by Lavin et al. showed that chewing a sweet pastille 10 times before swallowing compared to drinking a sweet liquid or water reduces intake (28). Another reason why we did not find a difference in intake after MSF might be because artificial sweeteners were used to manipulate sweetness of the model foods. It is contentious whether tasting (but not ingesting) artificial sweeteners has the same satiating capacity as glucose and sucrose (71). In addition, the fact that we did not see an effect of MSF on intake could be attributed to the time between MSF and the lunch; lunch was provided approximately 15 min after MSF because of the blood samples that were collected up to 10 min after MSF. This may have been too long to affect intake, which is confirmed by the appetite ratings that returned back to baseline 10 min after MSF.

To be able to manipulate texture and sweetness concentration while keeping all other food properties equal, model foods were used in this study. Although our model food system facilitates a very controlled way of studying the effect of small changes in food properties on physiological responses, the disadvantage of using model foods is that subjects are relatively unfamiliar with them, besides, our model foods were rated neutral for liking. Liking and expectations of food (ingestion) are both important to elicit a cephalic response, this could therefore be the reason why we did not find a cephalic insulin, PP or ghrelin response, in contrast with other studies (12, 30, 50, 72).

Another explanation why we did not find a response could be because larger responses are seen in overweight subjects while this study included healthy, normal weight subjects (54). In addition, 54% of the insulin curves in this study were responsive and 46% were non-responsive and because of that, on average, no effect of treatments could be found and no cephalic insulin response was seen.

Taken together the before mentioned food and subject prerequisites to evoke measurable CPRs, it can be concluded that these are highly exacting responses that do not occur in every person at every eating occasion.

## Conclusion

Our findings indicate that MSF on model foods does not lead to typical CPRs. Nevertheless, texture hardness and sweetness increases the total PP response, and MSF on hard texture increases the total ghrelin response compared to soft texture model foods. However, these effects are rather small and this study, among others, shows that there are major dissimilarities in cephalic phase endocrine responses to food stimulation between persons. This emphasizes that inter-individual factors need to be taken into account and stresses the importance of taking into consideration that there are cephalic responders and non-responders. These variable responses to food stimuli make it difficult to study the effect of realistic changes in food properties on CPRs. Therefore, more research is needed to further elucidate the effects of food texture and taste properties on CPRs.

## Ethics Statement

This study was carried out in accordance with the Declaration of Helsinki and approved by the Medical Ethical Committee of Wageningen University with written informed consent from all subjects.

## Author Contributions

ML, PS, MM, and CG designed the research (project conception, development of overall research plan, and study oversight). ML, PS, and MM wrote the manuscript. ML conducted the research. CG, PS, and MM read and approved the final version of the manuscript.

## Conflict of Interest Statement

The authors declare that the research was conducted in the absence of any commercial or financial relationships that could be construed as a potential conflict of interest.
